# Supplementary material for: Disposable ultrasound-sensing chronic cranial window by soft nanoimprinting lithography
Source: Nat Commun. 2019 Sep 19;10:4277. doi: 10.1038/s41467-019-12178-6 (PMC6753120; doi:10.1038/s41467-019-12178-6)
Supplement: Supplementary file 1 — Supplementary Information [file 41467_2019_12178_MOESM1_ESM.pdf]

Supplementary Information

Disposable ultrasound-sensing chronic cranial window by soft nanoimprinting lithography

Li et al.

Supplementary Note 1: Optimizing the sNIL process to manage the residual layer

Fabrication of usCCW via sNIL was accomplished by simply placing the PDMS soft mold on the surface of spin-coated PS layer. It allows for scalable fabrication of usCCW at significantly reduced cost, eliminating the needs for sophisticated instrument. However, the presence of residual layer with uneven thickness are the common problem found in the sNIL due to the absence of the high-pressure loading. The presence of the exceeding residual layer may interference the guided mode in the waveguide and thus, resulting in additional propagation loss. Although Reactive Ion Etching (RIE) process has been commonly used to remove the residual layer, we found it also unfavorably roughen the surface of the waveguide and results in the increased scattering loss. In this study, we have optimized the mold design to overcome this problem. As shown in Figure 1m and Figure 1n in the manuscript, the defined waveguide structure in the silicon mold is surrounded by recessed negative features with the finite width of 4.5 um. When being replicated in to the PDMS soft mold, those recessed features will then be replicated into the protruding pads surround the negative mold for waveguides (Figure 2p and Figure 2q in the manuscript). During the sNIL process, these recessed features shown in Supplementary Figure 1a are imprinted into the PS layer to define the waveguide structure shown in Supplementary Figure 1b. The finite width of 4.5 um significantly reduce the amount of the molten PS needs to be displaced and thus, effectively reduce the residual layer thickness. Supplementary Figure 1c shows the cross-sectional view of the fabricated waveguide structure encapsulated in the PDMS protection layer.

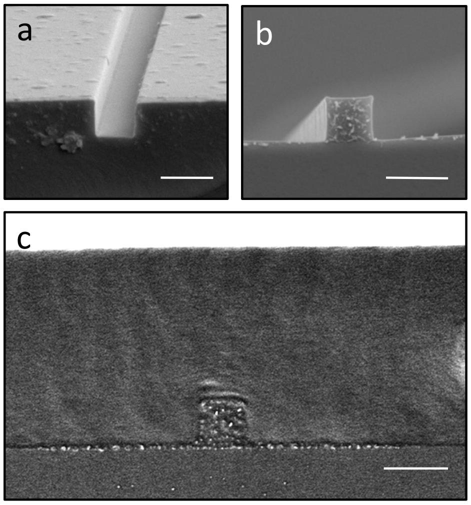

Supplementary Figure 1. SEM cross sectional images of (a) the PDMS soft stamp, (b) fabricated polymer waveguides using sNIL process, and (c) cross-sectional view of the fabricated waveguide structure encapsulated in the PDMS protection layer. Scale bars: 1 μm.

Supplementary Note 2: numerical simulation of optical waveguide modes

The polystyrene micro-ring resonator (MRR) waveguide was imprinted on a fused quartz coverslip (GE124, Ted Pella). The optical waveguide has a square-shaped cross-section with a side length of 800 nm. A 100-nm residual polystyrene layer on the quartz substrate was considered in the simulation. The optical mode profiles at 765 nm of the waveguide with and without protection layer were calculated numerically using finite element analysis (COMSOL Multiphysics), as shown in Supplementary Figure 2. In the calculation, the refractive indices of polystyrene, quartz, water, and PDMS are set to be 1.58, 1.46, 1.33, and 1.40[4], respectively.

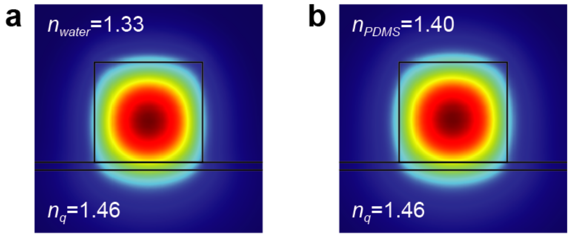

Supplementary Figure 2. Numerical simulation of optical waveguide modes (a) without and (b) with the PDMS protection layer.

Supplementary Note 3: calculation of acoustic reflection from PDMS surface.

The acoustic reflection R can be calculated by[5]

$$R = \left(\frac{Z_1 - Z_2}{Z_1 + Z_2} \right)^2, \qquad (1)$$

where Z_1 and Z_2 are the acoustic impedances of the materials in two sides of the boundary. In our case, Z_1 and Z_2 are 150 KRayl and 148 KRayl for water and PDMS, respectively[2]. The calculated R at the water-PDMS interface is 0.05%.

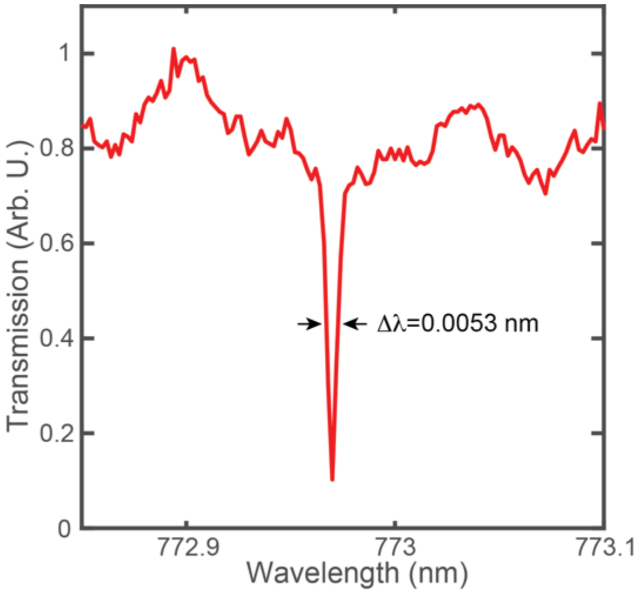

Supplementary Figure 3. Experimentally measured transmission spectrum with distinct resonance dip. The measured bandwidth of 0.0053 nm corresponds to a Q-factor of 1.46×10^5.

Supplementary Note 4: characteristics and performance of the MRR detector.

1. Q-factor of the optical resonance.

Experimentally measured transmission spectrum from the MRR is illustrated in Supplementary Figure 3. The measured resonance dip has a full-width-half-maximum (FWHM) bandwidth of 0.0053 nm, which corresponds to a Q-factor of 1.46×10^5.

2. The sensitivity and Noise equivalent pressure (NEP)

The sensitivity of MRR can be defined as:

$$Sensitity = \frac{dT}{dP} = \frac{dn_{eff}}{dP}\left(\frac{d\phi}{dn_{eff}}\frac{dT}{d\phi}\right)_{\phi_0}, \quad (2)$$

where P is the ultrasonic pressure; T is the transmission through the bus waveguide; n_{eff} is the effective refractive index; and ϕ_0 is the phase bias at the resonance wavelength. These three terms

dn_{eff}/dP, $d\phi/dn_{eff}$, and $dT/d\phi$ are collectively determined by the quality factor (Q-factor) of the MRR. With the experimentally measured Q-factor of 1.46×10^5 shown in the Supplementary Figure 4, the ultrasonic detection sensitivity of MRR is estimated to be 391.1 V/MPa. Considering the noise floor of 0.32 mV in our previous studies[6], the NEP is estimated to be 0.49 Pa.

3. Detection bandwidth of MRR detector

The detection bandwidth of MRR with and without the PDMS protection layer was measured experimentally. The photoacoustic signal was generated by focusing the 532-nm pulse laser (Elforlight SPOT-10-500-1064 with a BBO crystal for light frequency doubling, 10-nJ per pulse energy, 1-ns pulse duration), passing through the MRR, on to a 1.5-μm-thick carbon black thin film. The MRR detector was placed 1 mm away from the film to match the distance between usCCW and cortex.

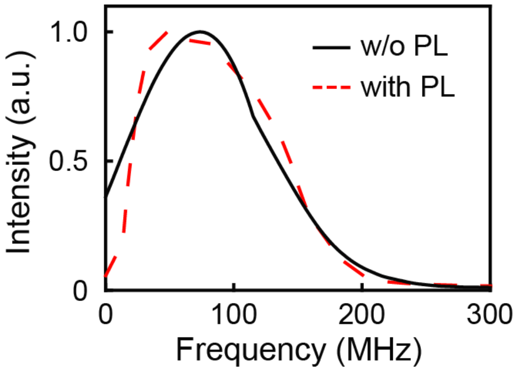

Supplementary Figure 4. Comparison of acoustic frequency responses of MRRs with and without PDMS protection layer (PL).

The calculated 6 dB bandwidths in Supplementary Figure 4 are 166.4 MHz for MRR without protection layer and 164.9 MHz for the one with PDMS protection layer. While our previous study demonstrated the MRR can operate at the broad frequency range from DC to over 250 MHz[7], in this study, the detection bandwidth is constrained by the limit operational bandwidth of low-noise

APD photodetector (Hamamatsu C4777) being used to convert the transmitted optical signals to the electric signals.

4. Imaging setup and procedures

As shown in Supplementary Figure 5, the PAM image on the cortex of an anesthetized mouse was taken under a commercial upright microscope (Olympus BX61). Photoacoustic signal was excited by a 532-nm Nd:YAG nanosecond pulsed laser (Elforlight SPOT-10-500-1064 with a BBO crystal for light frequency doubling, 10-nJ per pulse energy, 1-ns pulse duration). A dual-axis galvo-mirror assembly (Nutfield Technology) was used to raster scan the laser beam, and a matching 1:3 Keplerian telescope further coupled the laser beam to the back aperture of the objective lens (Olympus, 4X, NA 0.1) through the side port of the microscope body. The detection of the PA signal was accomplished by using the MRR ultrasound detector integrated on the CCW. To excite the MRR resonance, a narrow band continuous wave (CW) tunable laser (New Focus, TLB-6712, wavelength 765 nm to 781 nm) was coupled into the bus waveguide after passing through a fiber polarization controller, and then collected by a multimode fiber on the other end of the bus waveguide. The transmission intensity was interrogated by a photodetector (Newport, 2107-FC). Once the resonance was found, the wavelength was set at the wrist of the resonance dip. The output fiber was switched to an avalanche photodiode (APD, Hamamatsu c4777, bandwidth 10 kHz to 100 MHz). The signal was then amplified (Mini-circuits ZFL500NL+, 500-MHz bandwidth), digitized, and recorded by a Digitizer (CobralMax, GaGe).

During imaging, we placed the anesthetized mouse under the microscope and cleaned the CCW. The input fiber was linked to the CW tunable laser using a mechanical fiber splicer (Siemon, US-128 Ultrasplice) and the output fiber was connected to the photodetector or APD using a bare fiber

terminator. After imaging, we disconnected fibers and return the mouse back to the breeding cage. The mouse can move freely without breaking the fibers. Supplementary Movie 2 show a free-moving mouse wearing a usCCW.

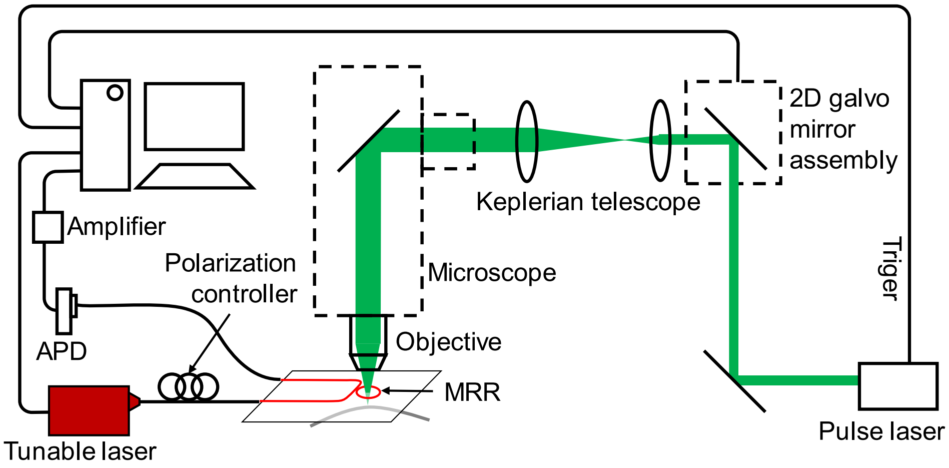

Supplementary Figure 5. Schematic of the imaging setup and data acquisition.

Supplementary Note 5: The background in high-resolution photoacoustic brain imaging.

The intact mouse skull has long been a major technical hurdle for high-resolution photoacoustic brain imaging. The underlying scattering of the incident laser beam and attenuation of acoustic signals due to acoustic impedance mismatch respectively hampers both lateral and axial resolution of photoacoustic imaging. Specifically, the strong scattering of the incident laser beam unfavorably broadens the focal spot size and thus reduce the lateral resolution. On the other hand, the large acoustic impedance mismatch at the skull-brain interface leads to strong attenuation of the generated photo-acoustic signals, especially the high-frequency signals above 30 MHz[1,2]. As a result, the best reported PAM imaging results has a highest axial resolution of 30 μm using a 50 MHz ultrasound transducer. Such a low axial resolution poses fundamental challenges in differentiating capillary networks in the skull, the durum, and the cortex. In a recent study by J. Yao et. al., it has found that "The skull degraded the image quality of PAM by blurring the optical focusing and attenuating the PA signal"[3]. In Yao's study, the intact mouse skull severely attenuated

the acoustic signals, resulting in a ~70% signal loss at 50 MHz acoustic frequency. Such a strong acoustic attenuation makes it almost impossible to further improve the axial resolution by increasing the ultrasound frequencies above 50 MHz.

To quantitatively illustrate the influence of the skull on the PAM image quality in adult mice, we include here the recently performed comparative studies of PAM brain image with and without the skull (Supplementary Figure 6). A ns laser (λ= 532 nm) at the pulse repetition rate of 30 kHz was used as the source of excitation. For the convenience, a 30 MHz ultrasound transducer was used to detect the generated photoacoustic signals. As shown in Supplementary Figure 6a, the PAM brain image was first performed in the adult mouse with intact skull, after surgically removing the scalp. The corresponding B-scan of dash line in Supplementary Figure 6a is shown in Supplementary Figure 6c. The strong optical scattering of incident ns-laser and the attenuation of the high-frequency ultrasound signals due to the presence of the skull unfavorably spoiled the recorded PAM brain images. For comparison, the skull of the same mouse was surgically removed, and the exposed region was subsequently covered with a transparent cranial window. Supplementary Figure 6b shows the acquired PAM image through the cranial window, with the imaging resolution significantly improved in comparison with the PAM image through the intact skull shown in Supplementary Figure 6a. Clearly, the use of cranial windows successfully eliminates scattering of the incident focused laser beam and thus, significantly improve the lateral resolutions of the acquired *en-face* PAM image (Supplementary Figure 6b). The improved optical focusing also results in stronger ultrasound signals through a cranial window (Supplementary Figure 6d), which is in clear contrast to the recorded ultrasound signals through the intact skull (Supplementary Figure 6c). Despite the greatly improved lateral resolution by eliminating the optical scattering, the axial resolution of PAM remains fundamentally limited to the ultrasound detection bandwidth. As shown in the x-z projection of the reconstructed images, the axial resolution with the cranial window (Supplementary Figure 6f) was only marginally improved in comparison with the intact skull (Supplementary Figure 6e).

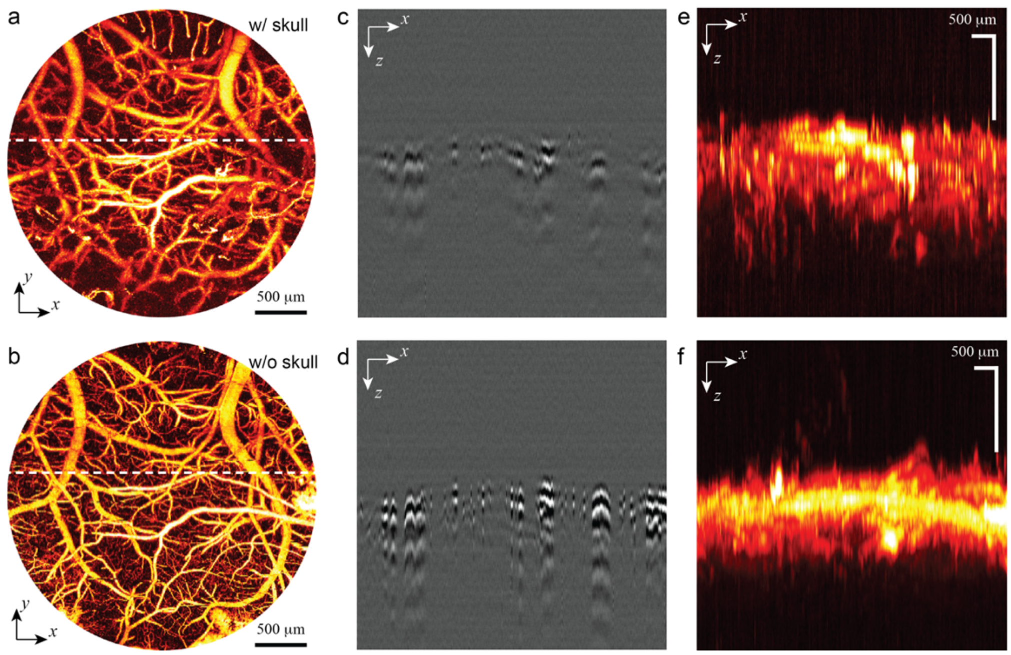

Supplementary Figure 6. The influence of the skull on the image quality is non-negligible in adult mice. A side-by-side comparison is shown below, where the PAM image acquired through the intact skull (a) shows much blurred microvasculature, in comparison to the acquired PAM image through a cranial window (b), due to the strong light scattering of the skull. (c) and (d) are the recorded B-scan ultrasound signals along the dashed lines marked in (a) and (b), respectively. The signals through a cranial window (d) are much stronger than through the intact skull (c). (e) and (f) are the x-z projection of the reconstructed B-scan PAM images shown in (a) and (b), respectively.

Supplementary Note 6: Long term monitoring of the cortical vasculature through a thinned-skull using usCCW.

In comparison with the PAM imaging in the open skull configuration, we also implanted usCCW on mice with thinned-skull. We found that thinned-skull configuration resulted in weaker ultrasound signal (see Supplementary Figure 7) due to higher acoustic attenuation caused by the residual bone structure. Moreover, the skull regrowth and window occlusion lead to further attenuations of both optical and ultrasound signals. We had to re-thin the skull after 20 days to minimize attenuations for longitudinal investigation.

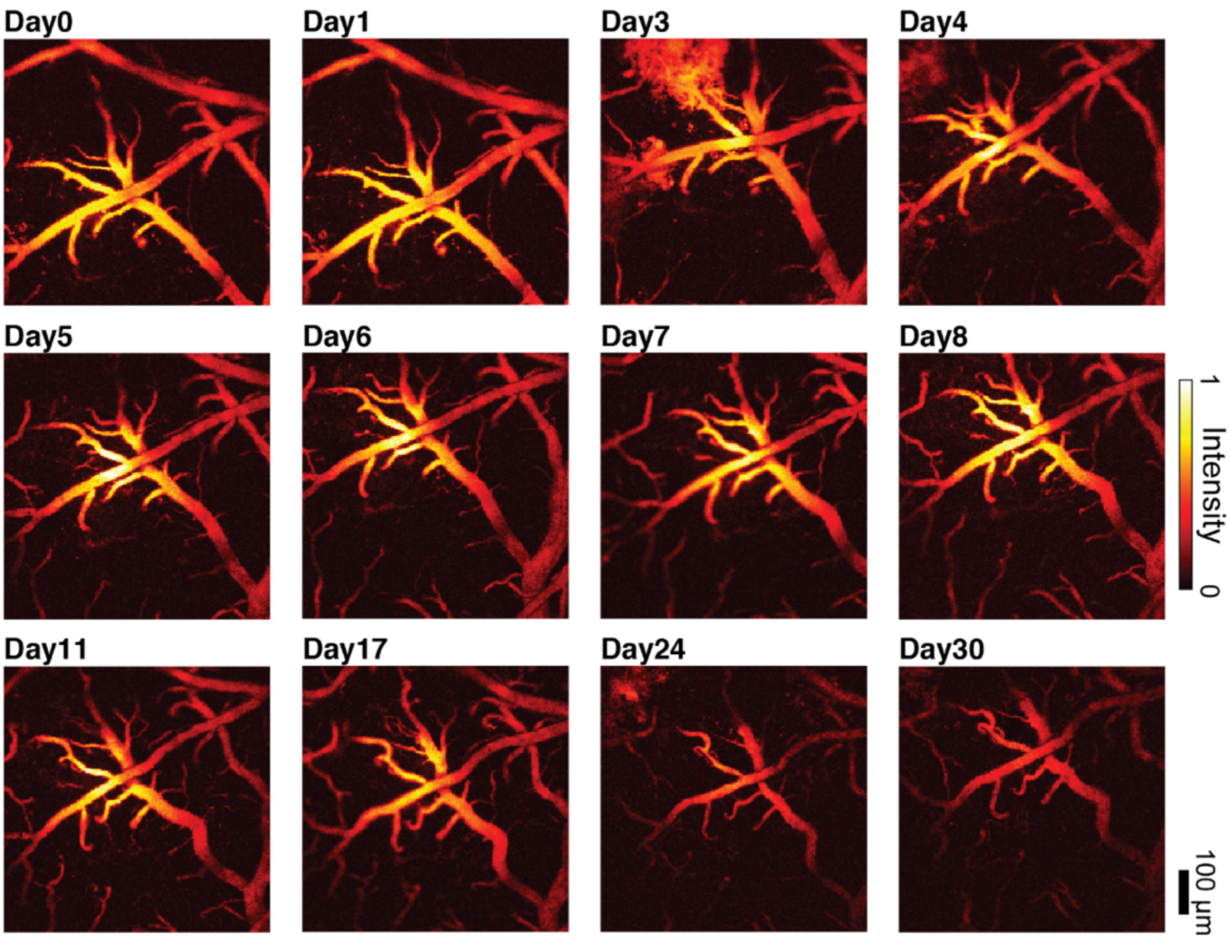

Supplementary Figure 7. Long term monitoring of the cortical vasculature through a thinned-skull using usCCW.

Supplementary References

1 Estrada, H., Rebling, J., Turner, J. & Razansky, D. Broadband acoustic properties of a murine skull. *Phys Med Biol* **61**, 1932-1946 (2016).

2 Kneipp, M. *et al.* Effects of the murine skull in optoacoustic brain microscopy. *J Biophotonics* **9**, 117-123 (2016).

3 Yao, J. *et al.* High-speed label-free functional photoacoustic microscopy of mouse brain in action. *Nature methods* **12**, 407-410 (2015).

4 Polyanskiy, M. N. *Refractive index database*, <https://refractiveindex.info>.

5 Sabri, F. *et al.* In Vivo Ultrasonic Detection of Polyurea Crosslinked Silica Aerogel Implants. *Plos One* **8**, e66348 (2013).

6 Li, H., Dong, B., Zhang, Z., Zhang, H. F. & Sun, C. A transparent broadband ultrasonic detector based on an optical micro-ring resonator for photoacoustic microscopy. *Scientific Reports* **4**, 4496 (2014).

7 Dong, B. *et al.* Isometric multimodal photoacoustic microscopy based on optically transparent micro-ring ultrasonic detection. *Optica* **2**, 169-176 (2015).
