# Supplementary material for: Disposable ultrasound-sensing chronic cranial window by soft nanoimprinting lithography
Source: Nat Commun. 2019 Sep 19;10:4277. doi: 10.1038/s41467-019-12178-6 (PMC6753120; doi:10.1038/s41467-019-12178-6)
Supplement: Supplementary file 3 — Description of Additional Supplementary Files [file 41467_2019_12178_MOESM3_ESM.pdf]

Description of Additional Supplementary Files

File name: Supplementary Movie 1

Description: Three-dimensional reconstruction of the PAM image of cortical vasculature in mouse brain *in vivo* visualizes the detailed vascular morphology.

File name: Supplementary Movie 2

Description: Video of a free-moving mouse in the breeding cage after surgical implantation of usCCW on its forehead. Only a pair of optical fibers is attached to the usCCW, which imposes minimal constraint to mouse's free-motion.
